# Supplementary material for: Navigating regulatory landscape: A qualitative exploration of medical devices and in vitro diagnostic medical devices oversight in Zimbabwe through key stakeholder perspectives
Source: PLoS One. 2024 May 16;19(5):e0287415. doi: 10.1371/journal.pone.0287415 (PMC11098438; doi:10.1371/journal.pone.0287415)
Supplement: Attachment — Submitted filename: Response to Reviewers.pdf [file pone.0287415.s002.pdf]

Charles CHIKU
419 Rydale Ridge Park,
Harare, Zimbabwe
E-mail address: charleschiku@gmail.com
+41783450910

16 September 2023

PROF KIM WARD
B.Pharm (UWC), MPharm (UWC), PhD (UWC)
School of Pharmacy
Private Bag X17
Bellville E-mail: kward@uwc.ac.za
University of Western Cape
7535
Tel: 021-959 3440 (w)
27-823797674 (mobile)

Response to Reviewers' comments for the study with the title Regulation of Medical Devices in Zimbabwe: A Qualitative Study with Key Stakeholders

Dear Prof Ward,

Thank you very much for your time and effort in reviewing the manuscript with the title on the subject line. I am grateful for the invaluable feedback you and the other reviewers provided to improve my work. I responded to each question raised during the review process. Where I needed to explain the rationale for the approach I used, I have done so as well. Each question had a corresponding response. The questions from the reviewers are indented and in bold to distinguish them from the responses provided.

Reviewers' Comments on the Introduction

Reviewers' comment: It would be useful to provide an overview of the maturity levels of medicines regulatory authorities in African countries, including Zimbabwe to get a general sense of the regulatory capacity.

Response: No maturity levels are published by national regulatory authorities in the context of medical devices, including In Vitro Diagnostic Medical Devices. The Global Benchmarking Tool + Medical Devices (GBT+MD) Revision VI+MD version, to evaluate the effectiveness of medical device regulation by national regulatory authorities, was published in April 2022. Therefore, when this version of the GBT +MD was published, there were no published data regarding the maturity levels of the African national regulatory authorities. The reviewer's comment is reasonable; however, based on the unavailability of published data regarding maturity levels, the only option was to include data obtained through a desk review survey conducted by the WHO and published on its website in 2016

(https://www.who.int/teams/health-product-policy-and-standards/assistive-and-medical-technology/medical-devices/regulations). Since Zimbabwe was the study setting, we have amended the wording to state where Zimbabwe stood regarding medical-device regulation. The statement reads, "*Zimbabwe was reported as regulating medical device market placement, but data is vague*".

Reviewers' comment: Line 62: What is meant by "no data"? Please clarify.

Response: A list of categories based on the availability of the basic elements of medical device legislation was described before stating the findings of the WHO survey. The following paragraph was added to explain the results of the survey,

" In 2016, the WHO conducted a survey utilizing a desk review to map the availability of legislation on the basic elements of medical devices: pre-market, market, and post-market in the African (Afro) region. Countries were categorized as follows:

- *Have all three types of elements,*
- *Have pre-market and placing on the market elements*
- *Have pre-market and post-market elements,*
- *Have placing on the market and post-market elements*
- *Have only pre-market elements*
- *Have only placing on the market elements*
- *Do not have any type of element*
- *Data not available (member states did not respond to the survey).*
- *Not applicable*

In the Afro region, 40% of countries have no medical device regulations, 32% have some, and 28% lack data. Globally, 58% of WHO members regulate these devices. Zimbabwe was reported as regulating medical device market placement, but data is vague (9). This regulatory disparity could lead to inferior devices and limit access to quality-assured ones."

Reviewers' comment: Line 82: Provide necessary references to support statements - unless the entire paragraph is entirely derived from reference 6?

Response: The statement was edited, and a reference was added to the support statements. Please refer to the response above.

Reviewers' Comments on the Methods section.

Reviewers' comment: It appears that no aspect of the methodology was piloted; is there a particular reason for this?

Response: The methodology has not been piloted for the following reasons.

- Very small sample size of the pool of participants. Piloting the methodology was not feasible because of concerns about diminishing the already limited participant pool.
- The study employed a non-complex methodology, using a questionnaire and semi-structured interviews. The questionnaire was adopted from a similar study conducted by Rugera et al., who sought to determine the regulation of medical devices in East African Community partner states, as stated in the Methodology section of the manuscript, and a reference was provided. This provided researchers with confidence in its implementation without a pilot phase.
- The study's methodology was built upon established regulatory practices that have been widely used and validated in previous research conducted by Rugera et al., and the researchers perceived the risk of significant issues arising as relatively low. (Rugera SP, McNerney R, Poon AK, Akimana G, Mariki RF, Kajumbula H, et al. Regulation of medical diagnostics and medical devices in the East African Community partner states. BMC Health Serv Res [Internet]. 2014 Oct 31 [cited 2021 Sep 18];14(1):1–7. Available from: https://bmchealthservres.biomedcentral.com/articles/10.1186/s12913-014-0524-2)
- Considering the study's exploratory nature using semi-structured interviews and its aim to generate preliminary insights rather than definitive conclusions, researchers might prioritize flexibility over rigorous piloting of semi-structured interview questions.

The following justification is added in the manuscript, " *The tool focuses on regulatory, pre-market, marketing, and post-marketing aspects. The small sample size prevented questionnaire testing and methodology piloting. However, our study used a questionnaire and interviews, bypassing the pilot phase and ensuring implementation confidence. Our study, grounded in regulatory practices, minimizes the risk of not being tested."*

Reviewers' comment: Line 99: Is there a particular reason why a legal expert from the medicines regulatory authority was not identified as a key informant? I imagine that the expert, technical knowledge of such a person would have been salient?

Response: We agree that a legal expert's knowledge of MCAZ would have been salient. However, during the process of data collection, legal experts were not keen to be interviewed, stating their involvement as purely legal in guiding the authority to structure and word laws and regulations to align with the national constitution. In terms of the development and amendment of laws and regulations, they were involved in that process, and the representatives felt that they were not competent enough to comment on the technical questions being asked in this study. Therefore, the results section recorded the legal expert as a non-respondent.

Lines 169-170 explain the inclusion of the legal department, " *Interviews with MCAZ Medical Devices and Microbiology unit and legal department provided insights into the IVD evaluation process. The sampling flowchart, which outlines the roles of the institutions, is depicted in Fig 1.*"

Reviewers' comment: Line 103: Was the data collection tool validated in this setting? If not, an explanation of why this may not have been necessary should be included.

Response: Please refer to the responses provided above to avoid piloting the methodology of this study. Additionally, the following statements were added to the manuscript to justify the non-piloting of the questionnaire,

" *The tool focuses on regulatory, pre-market, marketing, and post-marketing aspects. The small sample size prevented questionnaire testing and methodology piloting. However, our study used a questionnaire and interviews, bypassing the pilot phase and ensuring implementation confidence. Our study, grounded in regulatory practices, minimizes the risk of not being tested.*" (Lines 125-129)

Regarding the semi-structured interviews, the following justification was included in the methodology section of the manuscript (lines 157-159), "*The GBT indicators were accepted as the benchmark, so the questions were not piloted(14–16). The study's exploratory nature and semi-structured interviews prioritized flexibility over rigorous piloting of questions.*"

Reviewers' comment: Line 113: It is not clear if one staff member from each of the three institutions was approached or whether all were approached, and only three responded. If the former is correct, what was the rationale for including only one staff member from each agency, and what was the basis for selecting these individuals? Were there any efforts to triangulate findings if only one participant was included from each institution? Were the questionnaires self-administered or researcher-administered? Also, were the surveys completed electronically, telephonically or in person?

Response: The heads of the three institutions were approached and requested to provide experts from their respective agencies to complete the questionnaire. The heads of the institutions provided the names of the heads of the departments as people with the knowledge and expertise required to complete the questionnaire. Only one member of each institution completed the questionnaire because no new information emerged from the questionnaire, considering that the practices were based on the scope of work being conducted by each agency. Therefore, no efforts have been made to triangulate the responses from different respondents within the same agencies.

The questionnaires were self-administered. The respondents received the questionnaire by e-mail and sent the completed questionnaire to the researcher by e-mail. The following changes were made to the manuscript for clarity (lines 130-132), "*A yes/no questionnaire was completed by one staff member from each institution (MCAZ, MLCScCZ, and NMRL),*

covering topics like regulatory authority, legislation, safety principles, and post-market surveillance. The questionnaire was self-administered electronically and sent to the researcher. The study's standardized scope prevented triangulation.”

Reviewers’ comment: Line 100: Technically, the sampling approach combined a purposive and snowball method, correct? This should be clearly reflected.

Response: We agree with your comment. The statement has been amended to reflect the following (lines 165-170), *“ Purposive and snowball sampling selected participants, including representatives from medical device regulation institutions. Thirteen participants were chosen from MCAZ, MLCScCZ, and NMRL. Potential participants were identified using institutional input, regulatory documents, and local knowledge. Interviews with MCAZ’s Medical Devices and Microbiology unit and legal department provided insights into the IVD evaluation process. The sampling flowchart, which outlines the roles of the institutions, is depicted in Fig 1.*

Reviewers’ comment: Line 130: Is there a particular reason why the “GBT+Medical Devices” was not employed as the standard from which to develop the data collection tool? Please check the two references for the GBT, as they appear to be incorrect.

Response: The GBT was not employed as the standard to develop this study's data collection tool because it was considered a quantitative method. The GBT + Medical Devices and the PLOS ONE Journal (pre-print accessible at https://www. medrxiv. org/content/10.1101/2023.06.08.23291162v1) were considered for the quantitative manuscript that is under review. The two manuscripts are part of a Ph.D. dissertation that includes qualitative, policy analysis, and quantitative methods using GBT + Medical Devices. Considering the huge amount of work and the limitations of the journal, it was plausible to exclude the GBT and policy analysis from this manuscript under review. It made the GBT+ Medical Devices methodology a standalone manuscript.

The references were corrected as follows (Line 157-159),

“*The GBT indicators were accepted as the benchmark, so the questions were not piloted(13–15).”*

References:

13. Khadem Broojerdi A, Baran Sillo H, Ostad Ali Dehaghi R, Ward M, Refaat M, Parry J. The World Health Organization Global Benchmarking Tool is an Instrument to Strengthen Medical Products Regulation and Promote Universal Health Coverage. Front Med. 2020 Aug 19;7:457.

14. Guzman J, O’Connell E, Kikule K, Hafner T. The WHO Global Benchmarking Tool: A game changer for strengthening national regulatory capacity [Internet]. Vol. 5, BMJ Global Health. 2020. p. 3181. Available from: http://gh.bmj.com/

15. World Health Organization. Manual for benchmarking of the national regulatory system of medical products and formulation of institutional development plans. 2021;(February):1–81. Available from: https://cdn.who.int/media/docs/default-source/medicines/regulation-systems/benchmarking_manual_v2_09mar2021_clean.pdf?sfvrsn=33b0038d_5&download=true

Reviewers' comment: Line 134-135: These acronyms were introduced previously, and there is no need to write them out in full again.

Response. Agreed. The acronyms were corrected as follows, "The questionnaires were completed by representatives of the MCAZ, MLCScCZ, and NMRL."

Reviewers' comments: Line 162: Is there a reference to support the medical device advancements in SA and Tanzania?

Response: The statement was edited, and references were provided as shown below, *"Regulators from South African Health Products Regulatory Authority (SAHPRA) and Tanzania Medicines and Medical Devices Authority (TMDA) were interviewed to learn medical device regulation best practices and lessons in South and East Africa. The two countries were selected due to the progress in advancing their regulations(10,19,20)."*

10. Hubner S, Maloney C, Phillips SD, Doshi P, Mugaga J, Ssekitoleko RT, et al. The evolving landscape of medical device regulation in East, Central, and Southern Africa. Glob Heal Sci Pract [Internet]. 2021 Apr 1 [cited 2021 Nov 10];9(1):136–48. Available from: https://pubmed.ncbi.nlm.nih.gov/33764886/

19. Saidi T, Douglas TS. Medical device regulation in South Africa: The medicines and related substances amendment act 14 of 2015. South African Med J [Internet]. 2018 Mar 1 [cited 2022 Jan 11];108(3):168–70. Available from: https://doaj.org/article/bddc9501c446404ebb7aa554c5d42887

20. Keyter A, Salek S, Banoo S, Walker S. The South African Medicines Control Council: Comparison of Its Registration Process With Australia, Canada, Singapore, and Switzerland. Front Pharmacol. 2019;10(March):1–11.

Reviewers' comment: Table 1: Refer to comment for line 113. Additionally, were there only 12 participants who were approached for interviews or did some decline to be interviewed? There should be some indication of the response rate for the survey and for the interviews in paragraph where reference is made to Table 1.

Response: One respondent from MCAZ's legal department refused to participate in the interview. The following statement is included, "Twelve participants were interviewed, yielding a 92.3% response rate (one refusal)"(line 237).

Reviewers' comment: Line 185: Were the same researchers involved in interviews and transcribing? If not, what mechanisms were introduced to ensure the accuracy of the transcripts?

Response: One researcher was involved in the interviews, and verbatim transcription was used to ensure the accuracy of the transcripts. Verbatim transcription was chosen to capture participants' exact words, tone, and pauses. This level of detail is essential for analyzing nuances in responses. The following statement was added: *"Researcher (CC) introduced himself to participants, disclosing identity, credentials, occupations, gender, experiences, and training. To prevent bias, he avoided contact with participants before the interviews. Qualitative data were examined using a theoretical content analysis framework and NVivo 12. Interviews were transcribed verbatim to identify themes and concepts. Data were deductively coded using Rugera et al.'s frame and themes, with inductive codes added. CC and FB each coded four transcripts and then met to confirm and assess quality. Meetings for code review, idea sharing, and problem-solving were held. Open discussions and teamwork facilitated code agreement during discrepancies. We reached a consensus on these interpretations." (Line 201-208).*

Reviewer's comment: Line 191: Which coding frame was used for deductive coding? Elaborate on the manner in which inductive and deductive coding was used in tandem. This will enhance the reproducibility of the coding process.

Response: The coding frame was explained, and more details were provided in the following statement added to the manuscript, *"12. Interviews were transcribed verbatim to identify themes and concepts. Data were deductively coded using Rugera et al.'s frame and themes, with inductive codes added. CC and FB each coded four transcripts and then met to confirm and assess quality. Meetings for code review, idea sharing, and problem-solving were held. Open discussions and teamwork facilitated code agreement during discrepancies. We reached a consensus on these interpretations."*(lines 201-208)

Reviewers' comment: Line 193: The coding was not done in duplicate, correct? What then was the process for achieving consensus?

Response: Ensuring consensus included regular meetings, negotiations, and agreements between coders. The following statement was included to explain the process*: "Data were deductively coded using Rugera et al.'s frame and themes, with inductive codes added. CC and FB each coded four transcripts and then met to confirm and assess quality. Meetings for code review, idea sharing, and problem-solving were held. Open discussions and teamwork facilitated code agreement during discrepancies. We reached a consensus on these interpretations."(lines 201-208)."*

Reviewers' comment: Line 203: It makes sense to keep the interviewee's identity anonymous, but why/how was the "interview content" kept anonymous?

Response: The interviewees' identities were kept anonymous to ensure privacy and confidentiality. The following statement was added to state how the participants' identities were anonymized, *"Medical Research Council of Zimbabwe approved the study (MRCZ/A/2900), and participants could withdraw at any time with written consent. Interviews were confidential, and identities were protected. The consent form ensured anonymity. Real names were excluded from reports and publications. Interviews were anonymous, and participants were given codes. Codes were used for analysis and reporting. Interviews and documents were securely stored with restricted access, and digital files were password-protected and encrypted. This was done to maintain confidentiality and protect participants' identities."(Line 221-223).*

Results Section

Reviewers' comment: The configuration of the results should be reconsidered since there appears to be repetition of the same quotes – sometimes up to three times in results (table and text) and discussion (text). Consider summarising findings in the table and only presenting quotes in the results.

Response: The Results section was reviewed, and the most applicable quote and source were utilized to capture the summary of the theme. Tables 2 and 4 have been amended to summarize the findings.

Reviewers' comment: P12: The theme of limited regulatory capacity fails to mention essential capacity issues that would generally plague a low-income country such as lack of technical expertise, infrastructure and funding. These challenges feature under a different theme , namely, the "improvements to the system" theme on P15, but should also feature under capacity constraints. Again, if the findings in the table are summarised, as opposed to presenting selected quotes, this could provide a more complete picture and prevent repetition.

Response: The theme was reviewed and summarised in table 2, as shown below,

Limited regulatory capacity	The MCAZ and MLCScCZ did not have the technical expertise, funding and infrastructure to regulate medical devices, including IVDs. The NMRL lacked the technical expertise,

	competencies and reference materials to verify the safety and performance characteristics of IVDs to support the regulatory function assumed by the MLCScCZ.

Reviewers comment: P23-24: Formatting corrections are required in the table to align quotes with correct the correct interviewee.

Response: Table 4 was reviewed to include summary findings on the themes, and quotes were included in the results.

DISCUSSION SECTION

Reviewers' comments: Line 597: Clarify the type of dossiers referred to in this sentence. Is this for condoms and gloves?

Response: The sentence was reviewed to specify that dossiers are only for gloves and condoms. "*The MCAZ assessed glove and condom dossiers to validate manufacturers' claims.*" (Line 451)

Reviewers' comment: Line 694-708: It is important to reconcile this theory to the study findings in a short preface to this paragraph.

Response: The following summary has been added to link theory and the study's findings: " *A system-thinking approach involving practitioners, researchers, advocates, industry representatives, and leaders is crucial. Strategic stakeholder engagement, considering each group's needs, is necessary. Open communication and stakeholder participation in decision-making can enhance projects and relationships. Understanding system organization, dynamics, networks, and knowledge is vital for effective medical-device regulation." (lines 504-509).*

Reviewers' comment: Line 716-717: Italicize for consistency.

Response: The statements were italicized, and part was removed to reduce the number of words in the manuscript.

Reviewer's comment: Line 740: Was this "study" also initiated by the Tanzanian NRA? If so, this may make this explicit.

Response: This sentence has been deleted as it was misplaced and had no connection with the reference provided in the previous version of the manuscript.

Reviewer's comment: Figure 1: Provide a citation for this figure.

Response: The fig is now fig 2, and a citation is included.

Reviewers' comments: Figure 2: Is the roadmap novel in any way, or is it adopted entirely from the literature? If the latter, there should be a reference provided for the figure.

Response: The roadmap is not novel; a compilation of actions has been proposed to address the identified deficiencies. The compilation is based on the Global Model Regulatory and Regulatory Competency Frameworks. The citations of these two sources are included in the figure.

REVIEWERS COMMENTS FROM THE E-MAIL

Reviewers' Comment:1. Please ensure that your manuscript meets PLOS ONE's style requirements, including those for file naming. The PLOS ONE style templates can be found at

https://journals.plos.org/plosone/s/file?id=wjVg/PLOSOne_formatting_sample_main_body .pdf and

https://journals.plos.org/plosone/s/file?id=ba62/PLOSOne_formatting_sample_title_authors_affiliations.pdf

Response: The manuscript was formatted to meet the requirements stated in the PLOS One formatting and for the main body, title, and authors' requirements.

Reviewers' Comment 2: ***In your Data Availability statement, you have not specified where the minimal data set underlying the results described in your manuscript can be found. PLOS defines a study's minimal data set as the underlying data used to reach the conclusions drawn in the manuscript and any additional data required to replicate the reported study findings in their entirety. All PLOS journals require that the minimal data set be made fully available. For more information about our data policy, please see http://journals.plos.org/plosone/s/data-availability.***

Response: The minimal data set used to reach the conclusions drawn in the manuscript and any additional data required to replicate the reported study findings in their entirety will be made available in the Qualitative Data Repository to meet the PLOS One requirement. The draft data version is pending publishing in the Qualitative Data Repository, accessible at the link below pending curating.

https://data.qdr.syr.edu/dataverse/main

Reviewers Comment 5. Review Comments to the Author

Please use the space provided to explain your answers to the questions above. You may also include additional comments for the author, including concerns about dual publication, research ethics, or publication ethics. (Please upload your review as an attachment if it exceeds 20,000 characters.)

Response: The review response is 30,451 characters and will be uploaded as a separate attachment.

Reviewer's comment: Reviewer #1: This is a very thorough, well-executed and thoughtful study.

Response: Thank you for the compliment.

Reviewers's comment: General

•This is simply too long. Without tables it's 10593 words for a qualitative survey of 14 people. No one is going to read that. The authors are strongly advised to cut the text length in half.

•The authors could reduce some length and ease the reader through making use of an additional graphic and table. See below.

Response: The manuscript has been reviewed, and the text has been reduced to 6272, including the abstract, tables, and figure legends and captions.

METHODS SECTION

Reviewer's comment: Surveys (how many?) and semi-structured interviews (12) in Zimbabwe regulators.

Response: The statement was reviewed and read as follows, " *Between June and November 2022, we used purposive and snowball sampling techniques to select three participants from the Medicines Control Authority of Zimbabwe (MCAZ), the Medical Laboratory and Clinical Scientists Council of Zimbabwe (MLCScCZ), and National Microbiology Reference Laboratory (NMRL) completed the questionnaires. 12 participants representing regulators from these institutions were interviewed to understand the current status of medical devices, including IVD regulations and their relationships." (lines 122-129).*

Also interviewed (how many?) South African Health Products Regulatory Authority, Tanzania, and WHO to learn about "best practices in transitioning to medical device regulations."

Response: The statement was reviewed to read as follows, " *One participant from the South African Health Products Regulatory Authority (SAHPRA) and one from the Tanzania Medicines and Medical Devices Authority (TMDA) were interviewed to learn medical device regulation best practices and lessons in South and East Africa. The two countries were selected due to the progress in advancing their regulations(10,19,20)." (lines 170-174).*

Reviewers' comment: How was survey developed, tested, how were respondents identified and survey distributed? How many sent? How many received? How were interviewees identified?

Response: For the survey development, please refer to the response in the methodology section. Participants were selected as stated in lines 167-178, "*Thirteen participants were chosen from MCAZ, MLCScCZ, and NMRL. Potential participants were identified using institutional input, regulatory documents, and local knowledge. Interviews with MCAZ's Medical Devices and Microbiology unit and legal department provided insights into the IVD evaluation process. One participant from the South African Health Products Regulatory Authority (SAHPRA) and one from the Tanzania Medicines and Medical Devices Authority (TMDA) were interviewed to learn medical device regulation best practices and lessons in South and East Africa. The two countries were selected due to the progress in advancing their regulations(10,19,20). A WHO representative discussed the WHO Global Model Regulatory Framework for Medical Devices in the Afro region and its application in medical device regulations. The plan was to harmonize regional and international medical device regulations. The sampling flowchart, which outlines the roles of the institutions, is depicted in Fig 1."*

CONCLUSION SECTION*:*

Reviewers' comment: Accessibility is not the same concept as safety

Response: We appreciate that accessibility is not the same as safety. However, the goal of a regulatory system is to ensure that the population has access to medical devices, including IVDs, that are safe and of acceptable quality and performance. The conclusions drawn from this study are the same. Therefore, we decided to retain the statement.

INTRODUCTION SECTION

Reviewers' comment: Lines 40-46 are confusing and confused. These lines should be omitted, and making clear that the focus is on device manufacturer safeguards.

Response: We do not agree with the reviewer's comment that the regulatory system is concerned with manufacturer safeguards. Regulatory science examines the product lifecycle from design and development, verification and validation, risk classification, pre-market and post-market elements, based on meeting specified Essential Principles for Safety and Performance according to the Global Harmonisation Task Force and International Medical Devices Regulatory Forum guidance. The definitions of medical devices and In Vitro Diagnostic Medical Devices are provided to provide the context of this study. IVD medical devices are a subset of the medical devices. Therefore, we decided to keep the statement but reduce the wording.

Reviewers' comment: Lines 72-74. I like how the authors describe overall regulatory deficiencies and how Zimbabwe is inclusive. I like how they review other literature exploring this. It would be quite helpful to precede this with an explanation of what the different Zimbabwean regulatory offices/bodies are before delving into their deficiencies.

Response: A sentence was included to set the stage for the bodies responsible for some regulations in Zimbabwe. The following statement was added, *"MCAZ, an NRA, regulates medicines, condoms, and gloves as medical devices. MLCScCZ oversees medical lab professionals and operations, and evaluates IVDs related to HIV, TB, Malaria, and STIs for national tenders. NMRL, the reference lab, performs evaluations for market approval from the MLCScCZ." (lines 162-165)*

Reviewers' comment: Lines 75-76, the authors jump into describing stakeholder findings, before describing their research methods. This is out of place.

Response: The finding is not from the study conducted by the authors but from the study conducted by Ndomondo et al., and the reference is provided for the citation.

Reviewers' comment: Lines 80-83, the authors go from substandard and falsified medical devices, to manufacturer burden which are two separate concepts. The former is of primary responsibility, and the latter is of secondary interest. Further, I think a country with fewer regulations/oversight should not be equated with greater regulator burden for manufacturers.

Response: We agree that substandard and falsified medical devices and the manufacturer's burden to meet regulatory requirements are two different concepts; however, a regulatory system addresses both. The former was first introduced because the lack of a regulatory system exposes the country to substandard and falsified products. In countries where the regulatory system is not well-defined, the latter may require manufacturers to comply with regulations not harmonized with other countries where manufacturers meet different regulatory requirements. The statement does not equate a country with fewer regulations to one with greater ones. Therefore, the authors retained the following statement: "*Regulatory systems in the SADC region are inadequate to prevent, detect, and respond to substandard and Falsified medical products (12). This necessitates further investment in the existing frameworks, which should be a priority for the region. It is unclear if Zimbabwe followed this advice. Inadequate regulation can hinder manufacturers from meeting international approval standards, restricting access to needed devices. Regulatory bodies must ensure that medical devices, including IVDs, are safe, effective, and high quality to protect public health (13). We conducted a qualitative study to evaluate Zimbabwe's medical device regulation and gather stakeholder opinions on enhancement strategies." (lines 111-118).*

•Line 87: I don't think the authors are focused only on IVDs, are they?

Response: We have focused on medical devices, including IVDs. The statement was reviewed as *“Regulatory bodies must ensure medical devices, including IVDs, are safe, effective, and high-quality to protect public health (13)”* (lines 115-117)

Methods

•Line 103, Rugera requires a formal citation.

Response: The citation was added to follow the Vancouver style: "*The survey evaluated medical device safety and efficacy controls before and after marketing, using questions adapted from Rugera et al.'s study (14)”. (line 125)*

Reviewers’ comment: How were respondents identified? How contacted? By whom?

Response: The methodology section was reviewed to clarify the identification of the respondents, how they were contacted and by whom as follows, “ *Purposive and snowball sampling selected participants, including representatives from medical device regulation institutions. Thirteen participants were chosen from MCAZ, MLCScCZ, and NMRL. Potential participants were identified using institutional input, regulatory documents, and local knowledge. Interviews with MCAZ’s Medical Devices and Microbiology unit and legal department provided insights into the IVD evaluation process. One participant from the South African Health Products Regulatory Authority (SAHPRA) and one from the Tanzania Medicines and Medical Devices Authority (TMDA) were interviewed to learn medical device regulation best practices and lessons in South and East Africa.” (lines 166-174)*

Response:

•Line 113, “one staff member completed questionnaires…” goes in the results section

Response: This section has been moved to the Results section from the methodology section.

•Line 124: I don’t know what this means, as it is all about regulations.

Response: The terms have been defined to provide readers with the study's context. The statements were reviewed as follows,

“• The National Regulatory System is a legal framework that supports the regulatory system to guarantee medical product quality, safety, and performance.

• Market authorization involves issuing a licence or registration when medical products meet the conformity assessment standards.

• Vigilance is the practice of preventing, detecting, assessing, and understanding medical product-related issues to ensure quality, safety, and performance throughout a product's lifespan.

• Market Surveillance and Control ensure that products meet quality, safety, and performance standards when placed on the market.

• *A licensing Establishment ensures the quality, safety, and performance of medical products used in or exported from the country by licensing establishments in the product's value chain and life cycle.*

• *Regulatory Inspection audits establishments to guarantee compliance with laws, standards, norms, and guidelines.*

• *NRA laboratory testing verifies manufacturer performance claims for changes in pre-market approval or marketing authorization changes.*

• *Clinical Trials Oversight is the National Regulatory Authority's(NRA) legal responsibility to authorize, regulate, and end clinical trials..” (Line 140-156).*

Reviewers’ comment: Line 142: how many representatives were selected?

Response: The statement was reviewed to clarify that 13 participants from Zimbabwe were selected for the interviews, and in the results section, there was one refusal giving a response rate of 92.3%.

Reviewers’ comments: 134-144, very confusing. Would help readers to draw a flow map or a table with all the different regulatory agencies (without and outside of Zimbabwe), and what their regulatory purview is, such as pre-market, post-market etc. (which may have to go in the results section but can be referenced here.). There are so many acronyms in the paper, it’s very tedious to read without a visual aid.

Response: A flow chart was added, as Fig. 1 shows the different regulatory authorities, their purview, and the number of interviewed participants.

Reviewers’ comment: Lines184-185, who is the research team? How many designed, how many conducted, how many coded, how analyzed results?

Response: The author's contributions have been provided on page 1 of the manuscript.

RESULTS SECTION

Reviewers’ comment: Line 226, what’s the difference between a “participant “and a “critical stakeholder’

Response: The statement was corrected, “critical stakeholder was removed.” The statement was amended to *“Twelve participants were interviewed, yielding a 92.3% response rate (one refusal).” (line 237)*

DISCUSSION SECTION

Reviewers’ comments: lines 604-606. This is the first mention of classification schema. Would help to introduce this earlier than the discussion section.

Response: The schema was introduced briefly in the introduction section and reads, “Medical Devices are classified using a risk-based approach, ranging from Class A to D, based on the device's risk on the safety of the patient, user, public, and the environment. Class A is the lowest risk class, and class D has the highest risk (5). The level of scrutiny applied to medical devices during conformity assessment is used to evaluate whether the safety, performance, and quality of medical devices are proportional to the risk class of the device. Therefore, lower-risk devices are subjected to less stringent evaluation, whereas high-risk devices are evaluated using a more stringent assessment pathway (6).”

To reduce the number of words, the statement was rephrased in the introduction as follows, “ *Medical devices are classified from Class A (lowest risk) to Class D (highest risk) (5,6). Safety, performance, and quality evaluations are risk-class relative. Lower-risk devices face less strict assessments, while higher-risk ones undergo rigorous evaluations. Medical device regulations ensure safety and performance by mandating manufacturers follow risk control based on the device's risk category (7). While regulations must not hinder economic operators, devices must meet Essential Principles (EP) defined by the Regulatory Authority and detailed in the guidance documents of the Global Harmonization Task Force (GHTF) and International Medical Devices Regulatory Forum (IMDRF)’* (line 73-85).

Reviewers’ comment: Lines 694-707 should go into a table, along with other terms, to ease the readers.

Response: The definitions of the terms are presented in table format. Table 5 provides descriptions of these terms.

Reviewers’ comment: Lines 760-180 should go into a table.

Response: The recommendations were placed in a table format, as suggested. Table 6 lists the recommendations.

Reviewers’ comment: Line 814 “the main out of this study is a roadmap.” Where is it? Put it into a visual, please. The text is too tedious.

Response: The roadmap is shown in Fig 3. The figure has now been changed to a visual image.

I am very grateful for the opportunity to respond to the reviewers’ comments, which is an opportunity to improve the quality of the manuscript in the subject line. All the comments have been considered, resulting in a major overhaul of the manuscript, including reducing the word count to nearly half of the previous version. I am committed to addressing further concerns.

With kind regards,

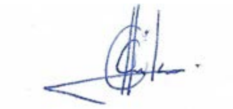

Charles CHIKU
