# Supplementary material for: Navigating regulatory landscape: A qualitative exploration of medical devices and in vitro diagnostic medical devices oversight in Zimbabwe through key stakeholder perspectives
Source: PLoS One. 2024 May 16;19(5):e0287415. doi: 10.1371/journal.pone.0287415 (PMC11098438; doi:10.1371/journal.pone.0287415)
Supplement: Attachment — Submitted filename: Responses to Reviewers comments .docx [file pone.0287415.s003.docx]

**Charles CHIKU**

**419 Rydale Ridge Park,**

**Harare, Zimbabwe**

**Email address: [charleschiku@gmail.com](mailto:charleschiku@gmail.com)**

**+41783450910**

**26 November 2023**

**Academic Editor**

**PLOS ONE**

**Response to Reviewers’ comments for the study PONE-D-23-17224R1**

**Regulation of medical devices in Zimbabwe: a qualitative study with key stakeholders.**

Thank you very much for your time and effort in reviewing the manuscript with the title on the subject line. I am grateful for the invaluable feedback you and the other reviewers provided to improve my work. I responded to each question raised during the review process. Where I needed to explain the rationale for the approach I used, I have done so as well. Each question had a corresponding response. The questions from the reviewers are indented and in bold to distinguish them from the responses provided.

**Reviewers Comment**

*Introduction:*

*- line 114- what is 'this advice' given to Zimbabwe?*

**Response:**

The advice was for member states in the SADC region to further invest in the existing frameworks to curb substandard and falsified medical devices. “*It was recommended that the member states in the SADC region further invest in existing frameworks, which should be a priority for the region to curb substandard and falsified medical products. It is unclear if Zimbabwe followed this recommendation.”(lines 120-122).*

***Reviewers Comment***

***Methods:***

***- line 130- yes/no questionnaire needs more explanation. is it to understand opinions, or is about existing structures/systems?***

Response:

The aim of surveying stakeholders in Zimbabwe was to comprehend medical device regulations in Zimbabwe and understand the current context, structures, experiences, challenges, and opportunities. This has resulted in the amendment of the statement: "From June to November 2022, using questionnaires and interviews, surveyed stakeholders to comprehend medical device regulations in Zimbabwe to understand the current context, experiences, challenges, and opportunities.” (lines 131-135).

***Reviewer’s comment:***

***- line 134- interviews seem to be 2 types- those with KIIs in Zimbabwe and those outside Zimbabwe. Were different interview guides used for each type of KII? What was the purpose of each and what were the broad themes/topics covered for each? Results seem to indicate that interviews in Zimbabwe were to understand the current context, experiences, challenges, opportunities etc while those internationally were to understand best practices, frameworks, recommendations. The methods need to be clear to understand and align with the results.***

Response:

The aim of surveying stakeholders in Zimbabwe was to comprehend medical device regulations in Zimbabwe to understand the current context, structures, experiences, challenges, and opportunities. This has resulted in the amendment of the statement: "From June to November 2022, using questionnaires and interviews, surveyed stakeholders to comprehend medical device regulations in Zimbabwe to understand the current context, experiences, challenges, and opportunities.” (Lines 131-135).

The explanation for interviewing regulators from South Africa, Tanzania and the World Health Organization was provided as shown below:

*“Furthermore, to understand best practices, frameworks, and recommendations for establishing and improving medical device regulations from other national regulatory authorities in the SADC region, semi-structured interviews were conducted with representatives from the South African Health Products Regulatory Authority (SAHPRA) and one from the Tanzania Medicines and Medical Devices Authority (TMDA). Additionally, a WHO’s Regulatory Systems Strengthening regulator was interviewed to gain perspective on using the Global Model Regulatory Framework for Medical Devices (GMRFMD) and how member states have been using it to establish and strengthen their medical device regulatory systems.” (lines 172-179).*

***Reviewer’s comment:***

***- line 166- suggested rewording- "Purposive and snowball sampling was used to select participants, ... "***

Response: The statement was reworded: "Purposive and snowball sampling was used to select participants, including representatives from medical device regulation institutions.” (lines 186-187).

***Reviewer’s comment:***

***- line 206- reference for Rugera et al.***

Response: The citation and reference for Rugera et al was added in line 229, “ Data were deductively coded using Rugera et al.'s frame and themes, with inductive codes added (14).”

***Reviewer’s comment:***

***- line 207-210- reword this to reflect the process of analysis and synthesis of qualitative data.***

**Response:** The section was reworded as follows, “Data were deductively analyzed and synthesized using Rugera et al.'s frame and themes, with inductive codes added (14) following an iterative process that involved making sense of the rich, non-numeric information gathered during qualitative research. To achieve this, the following steps were taken:

- Familiarization with the data involved transcribing information and immersing ourselves in its content.
- Coding of the data encompassed open coding, axial coding, and selective coding.
- Theme development comprised grouping codes into primary themes and breaking them into sub-themes.
- The constant comparison method involved ongoing scrutiny of the data to validate emerging patterns. Throughout this process, we documented our thoughts and reflections to facilitate the development of concepts and theories.
- Member checking, a validation step, entailed sharing our findings with participants to ensure that our interpretations aligned with their experiences and perspectives.
- Synthesis involved integrating individual themes and concepts to comprehensively understand medical device regulation in Zimbabwe and the broader region. This step aimed to construct a coherent narrative of the findings.
- Reporting was the final step, focusing on clearly articulating the findings. We utilized quotes and examples to illustrate key points and reflected on biases and their potential influence on the analysis.

CC and FB were involved in the synthesis and analysis process of the data.”(lines 228-247).

***Reviewer’s comment:***

***- line 213- "Participants were assigned anonymous codes ... " suggest using IDs instead of codes to not confuse with coding of data. And this sentence should be moved to the 'ethical considerations' section.***

Response: As suggested, " codes " were replaced with anonymous identifiers. Furthermore, the statement was moved to the ethical considerations section: "The consent form guaranteed anonymity, and real names were omitted from reports and publications. Participants were given anonymous identifiers based on their organizations (e.g., MCAZ KI07 for an MCAZ participant). Interviews were conducted anonymously, and these identifiers were used for analysis and reporting.” (lines 251-254).

***Reviewer’s comment:***

***- line 219- HOW were identities protected?***

Response: Identities were protected by data de-identification of the respondents by anonymizing by removing names and job titles of the participants. The statement has been edited as follows, “The consent form guaranteed anonymity, and real names were omitted from reports and publications. Participants were given anonymous identifiers based on their organizations (e.g., MCAZ KI07 for an MCAZ participant). Interviews were conducted anonymously, and these identifiers were used for analysis and reporting.*” (lines 259-261).*

***Reviewer’s comment***

***- Ethical considerations section- reorder, reword for improved readability.***

Response: The ethical considerations section was improved as follows, “The study (MRCZ/A/2900) received approval from the Medical Research Council of Zimbabwe. Participants had the option to withdraw at any time by providing written consent. During interviews, confidentiality was ensured, and identities were safeguarded. The consent form guaranteed anonymity, and real names were omitted from reports and publications. Participants were given anonymous identifiers based on their organizations (e.g., MCAZ KII7 for an MCAZ participant). Interviews were conducted anonymously, and these identifiers were used for analysis and reporting.

To maintain confidentiality, interviews and documents were securely stored with restricted access. Digital files were both password-protected and encrypted. This safeguarded participant identities by anonymizing identifiers and removing names and job titles from the data.”(lines 251-261).

***Reviewer’s Comment:***

- ***line 211- 3 themes mentioned are not aligning with how themes/results presented in results section. There seems to be 3 different analyses done- describe each separately (1) yes/no questionnaire, (2) KIIs within Zimbabwe, (3) KIIs outside Zimbabwe. Was Rugger's frame used for both types of interviews?***

Response: Thank you for raising this comment. This section was not very clear. Rugera’s framework was only used for the interviews conducted in Zimbabwe. This section has been moved to the results section since it was not in the right place in the previous version.

***Reviewer’s comment:***

***Results:***

***- line 224- results and discussion are 2 very separate broad sections.***

Response: Results and discussion were separated. The discussion was deleted, as shown in line 262.

***Reviewer’s Comment:***

***- include a few lines before presenting results to describe how the results are being presented, (1) yes/no questionnaire, (2) KIIs within Zimbabwe, (3) KIIs outside Zimbabwe.***

***- Present the Y/N questionnaire results in a table.***

Response: The introduction to the results section and how results will be presented was added as follows, “We present the study's findings that aimed to examine the medical device regulatory landscape in Zimbabwe and lessons learned from SAHPRA, TMDA, and WHO RSS in establishing and strengthening medical device regulations. The results are organized into three subsections, each addressing specific research questions. The first sub-section presents results from the questionnaires, the second from the semi-structured interviews of stakeholders in Zimbabwe, and the third from interviewing regulators from SAHPRA, TMDA and WHO RSS.” (lines 263-268).

***Reviewer’s comment***

- ***Table 2-***

***Themes listed here to not align with themes described in line 211- acts, regulations and guidelines.***

***The way the theme is named is presenting the result (ie. deleted limited and just have legal framework and regulatory capacity; delete 'the needs to improve the' and just leave it as medical devices regulatory system.Correct this in the subheadings of themes as well as in the table.***

Response: Themes in line 211 of the previous manuscript were deleted because they were not meant to be there. The themes have been corrected in lines 297-379 and in table 2 as shown below,

“Twelve participants were interviewed, yielding a 92.3% response rate (one refusal). Data were categorized into regulatory system components, revealing six main themes after transcript analysis: legal framework, regulatory capacity, conformity assessment, post-market surveillance, medical device regulation, and collaboration of regulatory authority.”

**Table 2: Primary Themes Arising from Interviews with Regulatory Stakeholders in the Medical Device Sector in Zimbabwe: A Study Spanning June to November 2022.**

| Theme | Summary Findings |
| --- | --- |
| Legal framework | The MCAZ lacked a legal basis to regulate medical devices. The MLCScCZ attempted to use the Health Professions Act to regulate IVDs, but it was not applicable. |
| Regulatory capacity | The MCAZ and MLCScCZ lacked technical expertise, funding, and infrastructure to regulate medical devices, including IVDs. The NMRL lacked technical expertise, competencies, and reference materials to verify IVD safety and performance, which the MLCScCZ was responsible for. |
| Conformity assessment | Glove and condom conformity assessment was identical despite different risk classes. |
| Post-market surveillance | No post-market surveillance is needed for public safety. |
|  |  |
| Medical devices regulatory system | Stakeholders agreed that a balanced, risk-based IVD regulatory system must be established and strengthened. Training and technical support, infrastructure, trained personnel, and transparency and accountability mechanisms are needed. |
| Collaboration of regulatory authorities | MCAZ, MLCScCZ, NMRL and other stakeholders must collaborate using systems thinking to ensure IVD regulations are risk-based, responsive, and effective. |

*Note.* MCAZ = Medicines Control Authority of Zimbabwe; MLCScCZ = Medical Laboratory and Clinical Scientists Council of Zimbabwe; NMRL = National Microbiology Reference Laboratory.

**Reviewer’s comment**

***Delete 'Stakeholders agree that'***

**Response**: Deleted as suggested.

**Reviewer's comment**

- lines 259-264- restructure for improved readability.

The statement was rephrased as, *“ The Health Professions Act, employed by MLCScCZ, lacks provisions pertaining to the oversight of In Vitro Diagnostic Devices (IVDS). Although the Medicines Control Authority of Zimbabwe (MCAZ) has drafted import/export regulations for medical devices, awaiting approval, it has also expressed intentions to amend the Medicines and Allied Substances Control Act (MASCA) to encompass the regulation of all health products. MCAZ contemplates a name change to reflect a broader scope beyond medicines, a matter that has been discussed within the Ministry of Health. The regulatory framework for In Vitro Diagnostic Devices (IVDs) under the National Microbiology Reference Laboratory (NMRL) remains ambiguous, with an interviewee acknowledging the difficulty in locating the specific Act of Parliament delineating NMRL's IVD regulatory functions (NMRL KI08)." (lines 317-324).*

***Reviewer’s comment***

***- line 268- reword "At the time of the interviews, ... "***

Response: Reworded as suggested, “ At the time of the interviews, the MCAZ only regulated condoms and gloves.” (line 328).

***Reviewer’s comment***

- ***line 278-279- the quote is not adding anything more than what is said at line 275.***

Response: The quote was deleted.

***Reviewer’s comment***

***- section on 'medical devices regulatory system' needs descriptive text to summarise this theme's findings and not just rely on quotes. The first quote on line 305 needs the pt ID included.***

Response: A descriptive text was added and read as, “ It was found that the medical devices regulatory system in Zimbabwe was not proactive, effective, or proportional to the risk class of medical devices. However, it is reactive to the disease burden if the disease is programmatically significant but without a proper regulatory framework based on legal provisions.” (lines 365-368)

The participant ID for the participant who commented was added as MLCScCZ KI12. (line 371).

***Reviewer’s comment:***

***- Table 4- are these themes or seemingly more of recommendations which are useful but suggest they be listed in a table of recommendations or summary of findings?***

Response: These are the best practices and lessons learnt from regulators outside of Zimbabwe. An introduction has been included to explain the process of analysis and generation of recommendations, “ WHO, SAHPRA and TMDA regulators were interviewed to learn best practices that can be used to make recommendations on the medical devices roadmap for Zimbabwe. Best practices obtained through interviews are typically reported as recommendations. Qualitative analysis of the data from interviews was used to generate themes representing patterns or recurring topics that emerge from the data. These themes were then used to inform the generation of recommendations. Insights were gained from these interviews, and interviewees' characteristics and themes are summarized in Tables 3 and 4.” (lines 391-395).

***Reviewer’s comment***

- ***headings lines 336 and 344- combine into one subheading of 'international standards/tools'***

Response: Headings below line 412 and line 419 are now combined under international standards and tools.

***Reviewer’s comment***

***- line 358- correct rep to be representative.***

Response: Rep was replaced with representative, as suggested by the reviewer in line 436 of the manuscript.

***Reviewer’s comment:***

***Discussion:***

***- there are alot of results presented in the discussion. This needs to be reviewed to restructure so that it is discussing rather than presenting results and in particular quotes (data) should not be in a discussion. Many are good quotes and better presented in the results. To improve the structure of the discussion, consider subheadings.***

Response: Quotes were removed from the discussion. Sub-headings were included in the discussion. The subheadings are Current Regulatory Landscape in Zimbabwe and Exploring Opportunities for Improvement.

***Reviewer’s comment***

***- references are missing at lines 486/487, 491.***

Response: The citation and references were added in lines 555-557, “Dube-Mwedzi et al. and Stanislav et al. identified inadequate capacity and effort in post-market surveillance, prevention, detection, and response to substandard and falsified medical products (12,25).”

***Reviewer’s comment***

***- lines 403-412- this section repeats itself (methodological difference) and would benefit from some editing to improve readability.***

**Response:** This section was rephrased to improve readability as follows, “Investigation of the regulatory landscape in Zimbabwe is meant to develop a roadmap for a medical device regulatory system to address the challenges and leverage the opportunities identified. The importance of medical device regulations upholds safety, quality, and performance standards for public health (8). Effective regulations necessitate a legal framework outlining the national regulatory authority's mandate, system, range of regulated products, and regulatory functions .

MCAZ drafted regulations for medical device imports and exports, but they are yet to be approved. MCAZ's lack of autonomy may explain the delay in approval.

Furthermore, the regulations lack legal definitions of products and entities to regulate (15).

Condoms and gloves met conformity assessment standards for approval. However, other medical devices like IVDs, microscopy stains, and veterinary devices are unregulated. Despite regulations, HIV, Malaria, TB, Syphilis, and COVID-19 IVDs are ineffective. Conformity assessment ensures safety and quality but not performance standardization. Due to limited capacity, IVDs' performance was partially verified without technical documentation, manufacturing site inspections, or labeling reviews. Thus, Zimbabwe does not have a formal medical device regulatory system, similar to the finding of Hubner et al. (10).” (lines 488-497).

***Reviewer’s comment***

***- lines 439- 441-,***

**Response:** This section was edited to improve readability: "The MLCScCZ acknowledged its inability to regulate IVDs and attributed this responsibility to MCAZ. However, despite possessing regulatory powers, MCAZ did not regulate IVDs, resulting in a regulatory gap. To address this, it was recommended that MCAZ take prompt action to enhance the regulatory framework. A unified regulatory approach was emphasized to streamline enforcement.

This challenge of IVD regulation was evident in a study by Dacombe et al. on HIV self-testing IVD regulation in Malawi, Zambia, and Zimbabwe. The study revealed confusion regarding the responsible regulator in Zimbabwe, with MCAZ and MLCScCZ claiming jurisdiction (11). To resolve this, MCAZ proposed the establishment of a single regulatory body for IVDs, with collaboration from MLCScCZ.

MLCScCZ expressed its intention to collaborate with MCAZ in IVD regulation by forming an advisory committee. Additionally, there is a need for clarity regarding NMRL's authority in conducting market authorization testing for IVDs. Legal measures are deemed necessary to empower NMRL to carry out testing for IVDs officially.” (lines 505-518).

***Reviewer’s comment:***

***- Table 5 has important elements to consider but rather than laying out the table from Systems Thinking in Tobacco Control, apply this to how it is or can be used in a system for regulation of medical devices in Zimbabwe. This has been done in Figure 2. Focus on describing figure 2 and table 5 is not necessary.***

**Response:** Table 5 was removed as suggested and the legend to fig 2 was expanded to explain the systems thinking approach in the context of medical devices regulation as follows, “Researchers, practitioners, leaders, and advocates are critical stakeholders in formulating and implementing medical devices, including IVD regulations. The integrated approach for effective regulation requires:

Systems Organization: The organization of systems aims to comprehend and advance collaborative frameworks responsible for the regulation, facilitation, management, and promotion of medical devices. This includes fostering systematic actions and facilitating continuous learning within the regulatory domain.

System Dynamics: System dynamics play a crucial role in modeling the complex interactions within the regulatory system. This involves capturing the intricacies of factors influencing medical device utilization, such as legislative frameworks, research endeavors, control activities, industry dynamics, and socio-cultural factors.

System Networks: System networks analyze collaborative relationships within the regulatory landscape. Their purpose is to enhance strategies for collaboration and minimize redundancy within the systems that oversee medical devices.

Systems Knowledge: System networks contribute to analyzing collaborative relationships, improving collaboration strategies, and mitigating redundancies within the broader context of medical device regulation.” (lines 584-594).

***Reviewer’s comment***

***- lines 575-580- line 578 is repeating line 576. could this roadmap be introduced earlier in discussion to describe the challenges and opportunities based on this roadmap.***

Response: As suggested, the roadmap was introduced at the beginning of the discussion: "Investigation of the regulatory landscape in Zimbabwe is meant to develop a roadmap for a medical device regulatory system to address the challenges and leverage the opportunities identified.” (lines 481-483).

***Reviewer’s comment:***

***- line 593- is data saturation necessary in a study of KIs/stakeholders describing the current context of a legal framework?***

***Response:*** The limitation was deleted.

***Reviewer’s comment***

***Conclusion:***

***-edit to improve readability. Currently, it reads like a bullet-point list***

Response: The conclusion was reviewed and read as follows,

“The findings from stakeholder interviews present a comprehensive examination of the existing landscape, frameworks, experiences, challenges, and prospects surrounding medical device regulation in Zimbabwe. Through a meticulous analysis of questionnaires and interviews with key regulatory entities, including MCAZ, MLCScCZ, and NMRL, several critical insights have emerged, shedding light on the current state of medical device regulation in the country.

**Legal Framework Challenges**

The study reveals a significant deficiency in Zimbabwe's legal framework for medical devices. While MCAZ utilizes the Medicines and Allied Substances Control Act to regulate condoms and gloves, a notable absence of comprehensive regulations encompassing all aspects of medical devices exists. This legal gap becomes particularly evident in the absence of laws mandating the safe and high-quality production of medical devices, leaving critical regulatory aspects unaddressed.

**Regulatory Capacity Limitations**

At the time of the interviews, regulatory entities, such as MCAZ, faced limitations in their capacity to regulate medical devices effectively. The study highlights challenges encountered by MCAZ in its efforts to regulate In Vitro Diagnostic (IVD) medical devices, prompting the engagement of MLCScCZ to establish an expert committee. Acknowledging MLCScCZ's difficulty regulating IVDs underscores the need for collaborative efforts and knowledge exchange between regulatory bodies.

**Conformity Assessment Issues**

Conformity assessment processes, particularly regarding the classification of medical devices, emerge as a critical concern. The absence of clear criteria for classifying medical devices and the incorporation of such criteria in draft regulations underscores the evolving nature of the regulatory landscape. As noted in the study, challenges in aligning conformity assessment with the risk class of medical devices further emphasize the necessity for well-defined and stringent assessment protocols.

**Post-Market Surveillance Deficiencies**

The study identifies deficiencies in post-market surveillance, with routine inspections being conducted for specific medical devices but not extending to formal surveillance. Acknowledging this weakness, particularly in the context of IVDs, emphasizes the need for robust post-market monitoring mechanisms to ensure the ongoing safety and efficacy of medical devices in the market.

**Reactive Regulatory System**

The regulatory system in Zimbabwe appears reactive rather than proactive, responding to specific disease burdens without a comprehensive framework based on legal provisions. The study participants agree on the inadequacy of existing regulations, focusing on specific diseases and leaving other categories of IVDs unregulated. Identifying this regulatory gap has prompted initiatives, such as drafting the Import and Export of Medical Devices regulations and IVD Medical Devices Regulations by MCAZ.

**Collaborative Opportunities**

Despite the identified challenges, there is a clear recognition of the importance of collaboration among regulatory authorities. Stakeholders, including MCAZ, MLCScCZ, and NMRL, are willing to collaborate, acknowledging each other's roles in the regulatory landscape. The emphasis on patient safety as a priority underlines the shared commitment to improving and strengthening medical device regulations through collaborative efforts.

In conclusion, the study underscores the multifaceted challenges within Zimbabwe's medical device regulatory framework and provides valuable insights for advancing the regulatory landscape. Regulatory bodies' identified gaps and collaborative intentions set the stage for future regulatory enhancements, emphasizing the imperative for a proactive, comprehensive, and legally sound approach to medical device regulation in Zimbabwe. The study's findings serve as a foundation for informed policy recommendations and actions to foster a robust and effective medical device regulatory environment in the country.” (lines 677-733).
