# Supplementary material for: Navigating regulatory landscape: A qualitative exploration of medical devices and in vitro diagnostic medical devices oversight in Zimbabwe through key stakeholder perspectives
Source: PLoS One. 2024 May 16;19(5):e0287415. doi: 10.1371/journal.pone.0287415 (PMC11098438; doi:10.1371/journal.pone.0287415)
Supplement: Attachment — Submitted filename: Responses to Reviewers comment R2.docx [file pone.0287415.s004.docx]

**Reviewer’s comment**

***Abstract
- Intro is 2 sentences. Need an additional 1-2 sentence to give background.***

Response:

The abstract was reviewed to give the background of the study as follows, *“However, there is a disparity in the availability of good quality and high-performing products due to the delay in the readiness of medical devices and IVD regulations in Low and Middle-Income Countries (LMICs), especially when compared to the progress made globally over the past 20 years global average.” (lines 36-39).*

***Reviewer’s comment***

***- It needs to be clear that different methods employed- a questionnaire and KIIs.***

The statement was reviewed to state the methods that were used for the various participants as follows, “*Between June and November 2022, we used purposive and snowball sampling techniques to select three participants from the Medicines Control Authority of Zimbabwe (MCAZ), the Medical Laboratory and Clinical Scientists Council of Zimbabwe (MLCScCZ), and the National Microbiology Reference Laboratory (NMRL) who completed the questionnaire. Twelve participants represented regulators from these institutions to understand the current status of medical device and IVD regulations and their relationships. Key informant interviews were conducted with three regulators from SAHPRA, TMDA, and the WHO RSS to learn the best practices for transitioning to medical device and IVD regulations to develop a roadmap for Zimbabwe.”* (lines 44-57).

***Reviewer’s comment:***

***- Analysis of questionnaires in main paper is descriptive analysis but not reflected in abstract.***

Response:

Analysis in the abstract was reviewed and reads as follows, “*We used descriptive analysis to analyze and examine data from questionnaires. Thematic approach and inductively developed a common coding framework to analyze emerging themes from interviews conducted in Zimbabwe and to generate recommendations from interviewing regulators from SAHPRA, TMDA, and WHO RSS”* (lines 57-58).

***Reviewer’s comment
- Abstract is too long and needs revision- consider condensing the conclusion as a start.***

Response:

The conclusion in the abstract was condensed as follows, “*The findings reveal significant deficiencies and gaps in the legal framework for regulating medical devices and IVDs, particularly highlighting the need for a legal framework and the absence of more comprehensive regulations. Regulatory entities like MCAZ face capacity limitations, especially in regulating medical devices and IVDs. Conformity assessment processes, medical device and IVD classification criteria, and post-market surveillance also represent challenges, highlighting the need for a well-defined framework and regulatory procedures. The Zimbabwean regulatory system pathway is characterized as reactive, prompting a collection of regulations on initiatives to address needs. Despite facing challenges, there is a recognition of the importance of collaboration among regulatory authorities, emphasizing a shared commitment to improving and strengthening medical device and IVD regulations for improved patient safety. By advocating for a proactive, comprehensive, and legally sound approach, indicating the potential for collaborations and synergies, the study provides a foundation for well-informed policy recommendations to guide enhancements and build a framework for a resilient, efficient and transparent regulatory environment in Zimbabwe and African region as a whole.”*  (lines 61-75). The abstract is still within the required 500 words limit.
***Reviewer’s comment
Intro
- Line 139- who recommended member states in the SADC region...?***

Response: The statement and citation were removed to reduce the introduction to literature that is specific for Zimbabwe.

***Reviewer’s comment
- Line 169- how was it sent back to researcher? “... and emailed back to the researcher”?***

The questionnaires were sent to the researcher by email. The statement was rephrased to read as follows, “The questionnaire was self-administered electronically and sent to the researcher by email.” (line 149-150).

***Reviewer’s comment***

***- Line 170- “study’s standardize scope prevented triangulation”- belongs in limitations section***

Response:

The lines was moved to the limitations section (line 688-689).

***Reviewer’s comment
Methods
- Line 208-211. Inset that these are based in Zimbabwe to make it clear.***

Response: These three institutions are based in Zimbabwe. (lines 212-213-195)

***Reviewer’s comment
- Line 216- separate paragraph when talking about interviews done outside Zimbabwe.***

Response:

A separate paragraph was created as suggested; please refer to line 220.

***Reviewer’s comment***

- ***Line 240- move to study procedures in first section of methods. How were data recorded? Audio recordings, notes... ?***

Response:

Moved to procedures in lines 203-205 and reads as follows, “Zoom and Teams were used for remote interviews. After consent, the data from interviews were recorded through audio recording.”

***Reviewer’s comment
- Lines 252-244 are not part of analysis. This is describing study procedures and should be moved to that section of the methods.***

Response:

The section was moved to the methods section in lines 201-203, “ *Researcher (CC) introduced himself during interviews to participants, disclosing identity, credentials, occupations, gender, experiences, and training. To prevent bias, he avoided contact with participants before the interviews.”*

**Reviewer’s comment
- Line 258-9- transcription and identifying themes and concepts are not interrelated. Move transcription to how data collected and processed. Then lines 257-260 should be restructured for improved readability and avoid repetition.**

Response: The concepts were separated. Transcription was moved to the methods section. The analysis was improved for readability from lines 252-267, *“Analysis of data from key informant interviews used a theoretical content analysis framework and NVivo 12. The analysis process consisted of deductive analysis supplemented by inductive codes in an iterative process. Key steps in this process included familiarization with the data, achieved through transcribing information and immersing in its content. Coding of the data encompassed open coding, axial coding, and selective coding. Theme development involved grouping codes into primary themes and breaking them into sub-themes.*

*The constant comparison method was applied, entailing ongoing scrutiny of the data to confirm emerging patterns. Thoughts and reflections were documented throughout this process to develop concepts and theories. Member checking, a validation step, involved sharing findings with participants to align interpretations with their experiences. Synthesis aimed at integrating individual themes and concepts to comprehensively understand medical device regulation in Zimbabwe and the broader region, constructing a coherent narrative of the findings.*

*The final step, reporting, focused on clearly articulating the findings. The author utilized quotes and examples to illustrate key points, while also reflecting on biases and their potential influence on the analysis. CC and FB were involved in the synthesis and analysis process of the data. ”*

***Revirewer’s comment:***

***- Line 267-271 talks about development of themes but its stated that themes come from Rugera’s themes. This needs to be clarified.***

Response: The line was clarified. It reads as follows, *“Analysis of data from key informant interviews used a theoretical content analysis framework and NVivo 12. The analysis process consisted of deductive analysis supplemented by inductive codes in an iterative process.”* (lines 252-257).

***Reviewer’s comment

Findings
- Survey results- A table of what exists and does not exist in the regulatory bodies would be beneficial.***

Response:

The table was included, comparing what exists and does not exist based on the WHO and IMDRF frameworks. See lines 313-315.

***Reviewer’s comment***

***- Table 2- no need to include the study duration in the title***

Response:

The study duration was removed. See line 345.

***Reviewer’s comment:
- Line 436- italicize the quote***

Response: the quote was italised, see lines 605-606.

***Reviewer’s comment***

***- Line 459 saying the same thing as previous sentence.***

Response: The repeated line was deleted. See line 436.

***Reviewer’s comment
- Line 460-462 should be moved to analysis section of methods.***

Response: The section was deleted as it was not adding value to the data analysis section. See lines 435-439.

***Reviewer’s comment
- Table 4- no need to include dates in the title***

Response: Dates deleted from the title (see line 433).

***Reviewer’s comment
- Line 481- ‘tolls’- should this be ‘tools’?***

Response: My apologies for the typographical error. It was corrected to read as tool/ (see line 604).

***Reviewer’s comment
- Line 510- is this the first time these 3 acronyms used?- need to write them out in full.***

Response: The statement was rephrased as follows, “Tanzania aligned its regulations with the regulations from the United States of America Food and Drug Administration (USFDA), the Australian Therapeutic Goods Administration (TGA)studied USFDA, TGA, and the Japanese Pharmaceutical and Medical Devices Agency(PMDA).” (lines 487-490).

***Reviewer’s comment***
***- Line 573, “despite...” this sentence is incomplete. Rephrase.***

Response: The statement was reviewed and reads as, “Despite the MLCsCZ registration of regulations, HIV, Malaria, TB, Syphilis, and COVID-19 IVDs through performance evaluations, the approach is ineffective.” (lines 629-632).

***Reviewer’s comment***
- Line 589- what is meant by ‘work’? be more specific.

Response: The statement was reviewed to read as, “ *Authorities in medical device and IVD regulation play a pivotal role in shaping policies, ensuring effective oversight, fostering collaboration, adapting to technological changes, and prioritizing public health. Their decisions directly impact the safety and efficacy of medical devices, as well as the overall well-being of patients and the healthcare system.”* (lines 692-695).

***Reviewer’s comment
- Line 638-9- rewrite for improved readability.***

Response: The statement was rephrased to read as follows, “*Reliance and recognition mechanisms involve an NRA deciding on product approval, even if it relies on other authorities. The relying authority is independent and accountable for decisions. Regulatory reliance can reduce access barriers and use resources efficiently. It can reduce uncertainties for innovators and improve crisis responses. The approach speeds up access to safe and quality health technology.”* (lines 729-734).

***Reviewer’s comment
- Line 657- “this finding is similar...” this can be merged into previous sentence to for improved readability.***

Response: The statement was merged as requested, *“Post-market surveillance documentation is lacking, with the MCAZ only monitoring condoms and gloves in the market. While the drafted regulations for medical device import and export and IVD include some post-market surveillance elements, they are not comprehensive. This aligns with the findings of Hubner et al. (10). Both Dube-Mwedzi et al. and Stanislav et al. have highlighted insufficient capacity and effort in post-market surveillance, specifically in the prevention, detection, and response to substandard and falsified medical products (24,25). Our study confirms a correlation with the findings of Stanislav and Mwedzi-Dube, though the studies were for the whole of Southern Africa, Zimbabwe included.” (lines 789-800).*

***Reviewer’s comment***

***- Lines 659 and 664 can be rewritten to merge and improve readability.***

Response: Please refer to the response above.

***Reviewer’s comment:
- Line 712- its not clear what this caption credit is for. Is it a reference for the requirements for effective regulation? If so cite it as any other reference.***

Response: The caption was adapted to medical device regulations. It was originally from the Tobacco Control Monograph. The caption was deleted and the citation was added in line 840.

***Reviewer’s comment:
- Line 734- “Table 6...” reword to be more clear. “...indicates gaps in MASCA identified from the South Africa and Tanzania experiences.”***

Response: The statement was rephrased to read as follows, “ Table 6 shows changes required in Zimbabwe's Medicines and Allied Substances Control Act based on the best practices learned from delineates divergences derived from the experiences in South Africa and Tanzania. , offering insights substantiating the proposed modifications to Zimbabwe's Medicines and Allied Substances Control Act (MASCA)..” (lines 865-869).

***Reviewer’s comment:
- Line 742-755 is giving a lot of results rather then discussing.***

Response: We agree with the reviewer’s comment. This section was deleted.

***Reviewer’s comment:***
- Line 773- “This framework covers...”- remove this. It’s a repeat of line 777.

Response: This section was deleted; see lines 841-842.

***Reviewer’s comment***

***Conclusion
- This is very long. Lines 797-836- are these another summary of what is in the discussion? Suggest to remove this while assuring that its already covered in the results/discussion.***

Response: We agree with the reviewer’s comment. Lines 794-840 were deleted.
