# Supplementary material for: Navigating regulatory landscape: A qualitative exploration of medical devices and in vitro diagnostic medical devices oversight in Zimbabwe through key stakeholder perspectives
Source: PLoS One. 2024 May 16;19(5):e0287415. doi: 10.1371/journal.pone.0287415 (PMC11098438; doi:10.1371/journal.pone.0287415)
Supplement: Attachment — Submitted filename: Responses to Reviewers.docx [file pone.0287415.s005.docx]

**NOTE: The line numbers refer to the “ Manuscript with Tracked Changes” in Simple Matkup Mode of Tracked Changes.**

**Reviewer #3: Abstract- this is still very long and needs to be shortened
Line 40- remove 'global average'**

*Response: “ Global average” was deleted. The abstract was reduced from 499* *to 355 words.*

**Reviewer’s comment**

**line 42- "opacity that is opaque"- this is not clear. consider rewording**

*Response: Rephrased and reads, "Zimbabwe's regulatory framework for medical devices and IVDs is unclear, leading to ineffective compliance and surveillance. Our study aimed to explore the current status of medical devices and IVD regulations in Zimbabwe.” (lines 37-39).*

**Reviewer’s comment
Line 43- "It is unclear if medical devices..." this is a repeat of the previous sentence. Suggest this sentence be removed.**

*Response: Deleted line 43.*

**Reviewer’s comment
Line 46- incomplete sentence. Add, "Additionally, we aimed to learn..."**

*Response, edited and reads in lines 41-50, “Semi-structured interviews were conducted with 12 regulators from the Medicines Control Authority of Zimbabwe (MCAZ) National Microbiology Reference Laboratory (NMRL), Medical Laboratory and Clinical Scientists Council (MLCScCZ) to understand the current status of medical devices and IVD regulations in Zimbabwe. Three participants completed a questionnaire to understand the regulatory landscape in Zimbabwe. Three key informant interviews were conducted with three regulators from the South African Health Products Regulatory Authority (SAHPRA), Tanzanian Medicines and Medical Devices Authority (TMDA), and World Health Organization Regulatory Systems Strengthening (WHO RSS) to learn best practices to create a roadmap for Zimbabwe.”*

**Reviewer’s comment
Line 49- 3 pts from 3 organizations is 9 people. Line 53 says 12 pts. Please double-check these numbers.**

*Response: The statement has been rephrased to correct the number of participants that were interviewed as follows, “Semi-structured interviews were conducted with 12 regulators from the Medicines Control Authority of Zimbabwe (MCAZ) National Microbiology Reference Laboratory (NMRL), Medical Laboratory and Clinical Scientists Council (MLCScCZ) to understand the current status of medical devices and IVD regulations in Zimbabwe. Three participants completed a questionnaire to understand the regulatory landscape in Zimbabwe. Three key informant interviews were conducted with three regulators from the South African Health Products Regulatory Authority (SAHPRA), Tanzanian Medicines and Medical Devices Authority (TMDA), and World Health Organization Regulatory Systems Strengthening (WHO RSS) to learn best practices to create a roadmap for Zimbabwe.” (lines 41-50)*

**Reviewer’s comment
line 58- "thematic approach" sentence needs to be restructured. "Using a common coding framework, we used thematic analysis for interviews conducted in Zimbabwe and with international regulators."**

*Response: The statement has been amended to “We analyzed qualitative data using a thematic analysis” (Lines 50-51).*

**Reviewer’s comment
Methods:
Table 1 goes in results**

*Response:*

*Table 1 was moved to the results section. (See lines lines 282-284).*

**Reviewer’s comment
line 255- NVivo does not analyze. It is used top code and organize data. Move mention of its use in line 258, "Coding of data... and selective coding, using NVivo 12."**

*Response:*

*The phrase has been amended in lines 245-247: " NVivo 12 software (QSR International) was used for data coding, encompassing open, axial, and selective coding. Theme development involved grouping codes into primary themes and breaking them into sub-themes.”*

**Reviewer’s comment
Line 276- you discuss anonymity, yet the participants' organization and their job roles are listed. This is very identifying. In line 282, its say removing job titles from data. Yet the job title is in Tables 1 and 4. Please address this important ethical issue.**

*Response:*

*We agree with the reviewer’s comment. We have removed participants' job titles from Tables 1 and 4 to address the ethical issue. The Ethical consideration section was also amended to read, “Ethics approval was obtained from the Medical Research Council of Zimbabwe, approval number (MRCZ/A/2900). All the participants gave written informed consent to take part in the interviews. Names used in this study are pseudonyms.” (Lines 260-262)*

**Reviewer’s comment
Results:
Table 2- shorten the questions to be key phrases/points.**

*Response:*

*The questions have been changed to phrases; please refer to Table 2 (lines 301-3023).*

**Reviewer’s comment
Discussion:
line 568- i believe this is first time RLS is used. write it out in full in the section 'exploring opportunities for improvement' there are still results presented in the form of quotes. This discussion needs to be more tidy by summarising and discussing results rather than presenting them.**

*Response:*

*RLS was replaced with LMIC, first mentioned in line 37 in full. Please refer to line 558. Quotations were summarised and removed from the discussion.*

**Reviewer’s comment**
**I recommend that authors have someone external to the project review this paper in detail. There is still some room for improvement in the writing style to be more concise and scientific.**

*Response:*

*A reviewer who published a number of papers reviewed the paper to improve its style and readability, including its scientific approach. His profile is accessible at* [*https://www.lshtm.ac.uk/aboutus/people/timire.collins*](https://www.lshtm.ac.uk/aboutus/people/timire.collins)*.*

**Reviewer’s comment:**

**Reviewer #4: Thank you for the opportunity to review this interesting article which looks at medical device regulations in Zimbabwe and Tanzania. The article includes recommendations for further maturity of regulations and regulatory structures in these countries, and potentially these are applicable more widely in similar settings.**

**Firstly, I would like to check with the authors that they have explicit consent for the details on job title used here and especially in table 4 this is quite an identifying characteristic (just short of naming participants in many cases).

I have a number of other, minor comments, with the hope to improve readability and therefore bring forward the main learning of this manuscript.**

**Introduction
Line 82: “It also includes products…” what is “it” referring to?**

*Response:*

*“It” refers to medical devices, carrying over from the previous statement. The statement has been reviewed to read as, “Medical devices also include products that affect the structure or any physiological process within the human body, provided they do not achieve their principal intended action by pharmacological, immunological, or metabolic means.” (see lines 80-83).*

**Reviewer’s comment
Line 104: please introduce the IMDRF acronym here and remove full name from line 109.**

*Response: The statement has been reviewed accordingly. The statement reads as follows, “Medical devices and IVD guidance for regulations set at the international level by the International Medical Devices Regulators Forum (IMDRF) were designed to ensure that the manufacturer who places the product on the market is responsible for all activities, safety and performance by mandating manufacturers to follow risk control based on the device's risk category (8). While regulations must not hinder economic operators, devices must meet the Essential Principles defined by the regulatory authority and detailed in the Global Harmonization Task and IMDRF guidance documents” (lines 101-107).*

**Reviewer’s comment
Line 114: I have not seen the African region referred to as the “Afro” region – is this common? Perhaps use “African region” instead.**

*Response: “Afro” is normally used by the WHO. However, the line has been amended to read as African region: "In Africa, 40% of countries had no medical devices regulations, 32% had some, and 28% lacked data.” (lines 111-112).*

**Reviewer’s comment
Line 119-120: Reference for this?**

*Response:*

*The reference has been added as shown in line 119 as follows, “Zimbabwe, a low-income country in Southern Africa, has inconsistent medical device and IVD regulations (11).”*

**Reviewer’s comment:
Methods
General – authors state that COREQ is used for reporting however several things are left out: what are the interviewers' experience and training? what is the relationship, if any, between the interviewer and the participants? Any prior knowledge?**

*Response:*

*The section has been reviewed to add more details. The section reads, “The researcher (CC) identified himself to participants during interviews, disclosing his identity, qualifications, occupations, gender, background, and training. The researcher did not have a relationship with the participants. As a Regulatory Affairs Professional for Medical Devices and IVDs with five years of experience in the field, the interviewer conducted remote interviews using Zoom or Teams. The researcher explained the purpose of the study to the participants before obtaining informed consent to take part in the study and to use an audio recorder. All the in-depth interviews were conducted in English, audio recorded, and transcribed verbatim.” (Lines 195-202).*

*The other aspects relating to the study context, participant recruitment, participant characteristics, interview guide, data collection procedures, ethical consideration, data analysis, and researcher reflexivity findings, including quotations, are already included in the study.*

**Reviewer’s comment
Line 143-145: The sentence that begins with “However, our study…” belongs in limitations section. Also I don’t fully understand this, please rephrase for clarity.**

*Response:*

*The statement has been rephrased and moved to the limitations section: "The tools used in the studies were not piloted due to the small sample size, which prevented questionnaire testing and methodology piloting. Additionally, our study used a questionnaire and interviews to bypass the pilot phase and ensure implementation confidence. Our study is grounded in well-established regulatory practices, minimizing the risk of not being tested.”(lines 695-699)*

*The statement was added to explain why the data collection tools were not piloted before the study.*

**Reviewer’s comment
Medical devices and IVD regulatory system
Line 392 – 394 – regarding the sentence that begins “However, it is reactive…” I see that the further text below is an example of what you mean by this, so perhaps you don't need this sentence? If you do decide to keep it, please rephrase it to add clarity.**

Response:

The statement was deleted and amended. It reads, ***“****It was found that the medical devices and IVDs regulatory system in Zimbabwe was not proactive, effective, or proportional to the risk class of medical devices and IVDs.”* (line 376).

**Reviewer’s comment
International standards and tools
Line 442 – On first reading, I was wondering what the participant mean that the model was updated? Now I see there is a section currently in the discussion (Line 630 – 659 and Table 5), which if it is indeed part of what you found in the study, is results and should be moved up here specifically as it helps to explain this paragraph and statement by the participant.**

*Response. The participant indicated that there were discussions to update the framework to include indicators of the GBT into the framework. The quotation from the South African participant was removed. The statement has been amended to read, “There are plans to revise the WHO GMRFMD. The WHO representative said, “The GBT indicators prompted discussions to update the model to include GBT + medical indicators”(WHO KI13).” (lines 427-428).*

*Line 630 in the previous version of the manuscript has been amended to remove the quotes and discuss the significance of streamlining the regulatory framework as follows, “In the manuscript's discussion, it is imperative to acknowledge the crucial role of stakeholder involvement in achieving regulatory harmonization. The participation of policymakers, regulators, and economic operators is indispensable for garnering regulatory acceptance. Harmonization and convergence of medical device regulations, as seen in South Africa and Tanzania, are vital. Convergence adopts international guidelines, while harmonization standardizes technical guidelines, easing the regulatory load on manufacturers and regulators (28)” (lines 619-626).*

*We think it is inappropriate to include Table 6 in the results section. The table is a compilation of recommendations from the findings in evaluating Zimbabwe's current medical devices and IVD regulations and best practices learnt from SAHPRA and TMDA. Therefore, we have decided to maintain it in the discussion section of the manuscript.*

**Reviewer’s comment
The transition period to implement new regulations
Line 489: what does “rely on the status” mean?**

*Response:*

*The quotation was incomplete. It has been corrected to refer to the previous regulatory approval by the PHLB, which was not stringent compared to the one implemented when new regulations were enacted. “ “Initially, the population had to rely on the previous regulatory approval by the PHLB before full registration was allowed. Although grandfathering was acknowledged, regulations covered all aspects, including site performance” (lines 480-482).*

**Reviewer’s comment
Line 491-2: Please clarify whose statement this is - the authors of this manuscript or the participants. If this is the authors’ statement, it belongs in the discussion.**

*Response: The statement belongs to the author. It was deleted from the results section. Please refer to lines 478-479.*

**Reviewer’s comment
Discussion
Line 630 – 659 and Table 5 – I think this belongs in the results section.**

*Response:*

*The section was edited and reads, “In the manuscript's discussion, it is imperative to acknowledge the crucial role of stakeholder involvement in achieving regulatory harmonization. The participation of policymakers, regulators, and economic operators is indispensable for garnering regulatory acceptance. Harmonization and convergence of medical device regulations, as seen in South Africa and Tanzania, are vital. Convergence adopts international guidelines, while harmonization standardizes technical guidelines, easing the regulatory load on manufacturers and regulators (28)” (lines 619-626).*

**Reviewer’s comment**
**Line 638 – “Table 6” is below labelled table 5.**

*Response:*

*The table was amended to Table 6. We think it is inappropriate to include Table 6 in the results section. The table is a compilation of recommendations from the findings in evaluating Zimbabwe's current medical devices and IVD regulations and best practices learnt from SAHPRA and TMDA. Therefore, we have decided to maintain it in the discussion section of the manuscript.*
