# Supplementary material for: Navigating regulatory landscape: A qualitative exploration of medical devices and in vitro diagnostic medical devices oversight in Zimbabwe through key stakeholder perspectives
Source: PLoS One. 2024 May 16;19(5):e0287415. doi: 10.1371/journal.pone.0287415 (PMC11098438; doi:10.1371/journal.pone.0287415)
Supplement: Attachment — Submitted filename: Reviewer comments _07.07.23.pdf [file pone.0287415.s001.pdf]

PROF KIM WARD
B.Pharm (UWC), MPharm (UWC), PhD (UWC)

Postal address:
School of pharmacy
Private Bag X17
Bellville
University of Western Cape
7535

Tel: 021-959 3440 (w)
27-823797674 (mobile)

E-mail: kward@uwc.ac.za

---

03 August 2023

Reviewer report
Reviewer: Prof Kim Ward
Journal: PLOS ONE

Title: Regulation of medical devices in Zimbabwe: A qualitative study with key stakeholders

General Comments

This relevant and timely study sheds light on the medical device regulatory landscape in a low-income country. The regulation of medical devices is critical to improving access to safe and reliable health products. The insights from key informants from the Medicines Control Authority, the Medical Laboratory and Clinical Scientists Council and the National Microbiology Reference Laboratory reveal both a lack of regulation as well as fragmented oversight involving these three agencies in Zimbabwe. This paper could potentially serve as a catalyst to improve engagement and communication between the three national institutions to create more robust and streamlined regulation of medical devices. The "best practice" perspectives from South Africa, Tanzania and the WHO highlight key enabling factors or facilitators towards developing a legislative framework for medical devices and for the successful implementation of medical device regulation.

The study design, sampling, data collection and data analysis methods are all suitable to address the research questions, although in some cases more detail is required to ensure transparency and reproducibility. Additionally, mechanisms for ensuring accuracy should be adequately explained.

The manuscript is generally well written, however, a number of language and typographical errors were found throughout the manuscript (e.g. line 157-159, line 165-166, line 240, line 496-498, line 816). The in-text citations should also be checked against the final list of references.

Introduction

It would be useful to provide an overview of the maturity levels of medicines regulatory authorities in African countries, including Zimbabwe to get a general sense of the regulatory capacity.

Line 62: What is meant by “no data”? Please clarify.

Line 82: Provide necessary references to support statements - unless the entire paragraph is entirely derived from reference 6?

Methods

It appears that no aspect of the methodology was piloted; is there a particular reason for this?

Line 99: Is there a particular reason why a legal expert from the medicines regulatory authority was not identified as a key informant? I imagine that the expert, technical knowledge of such a person would have been salient?

Line 103: Was the data collection tool validated for this setting? If not, an explanation of why this may not have been necessary should be included.

Line 113: It is not clear if one staff member from each of the three institutions was approached or whether all were approached and only three responded? If the former is correct, what was the rationale for including only one staff member from each agency and what was the basis for selecting these individuals? Were there any efforts to triangulate findings if only one participant was included from each institution? Were the questionnaires self-administered or researcher-administered? Also, were the surveys completed electronically, telephonically or in-person?

Line 100: Technically, the sampling approach combined a purposive and snowball method, correct? This should be clearly reflected.

Line 130: Is there a particular reason why the “GBT+Medical Devices” was not employed as the standard from which to develop the data collection tool? Please check the two references for the GBT as they appear to be incorrect.

Line 134-135: These acronyms were introduced previously and there is no need to write them out in full again.

Line 162: Is there a reference to support the medical device advancements in SA and Tanzania?

Table 1: Refer to comment for line 113. Additionally, were there only 12 participants who were approached for interviews or did some decline to be interviewed? There should be some indication of the response rate for the survey and for the interviews in paragraph where reference is made to Table 1.

Line 185: Were the same researchers involved in interviews and transcribing? If not, what mechanisms were introduced to ensure accuracy of the transcripts?

Line 191: Which coding frame was used for deductive coding? Elaborate on the manner in which inductive and deductive coding was used in tandem. This will enhance the reproducibility of the coding process.

Line 193: The coding was not done in duplicate, correct? What then was the process for achieving consensus?

Line 203: It makes sense to keep the interviewee’s identity anonymous but why/how was the “interview content” kept anonymous?

Results

The configuration of the results should be reconsidered since there appears to be repetition of the same quotes – sometimes up to three times in results (table and text) and discussion (text). Consider summarising findings in the table and only presenting quotes in the results.

P12: The theme of limited regulatory capacity fails to mention essential capacity issues that would generally plague a low-income country such as lack of technical expertise, infrastructure and funding. These challenges feature under a different theme , namely, the “improvements to the system” theme on P15, but should also feature under capacity constraints. Again, if the findings in the table are summarised, as opposed to presenting selected quotes, this could provide a more complete picture and prevent repetition.

P23-24: Formatting corrections are required in the table to align quotes with correct the correct interviewee.

Discussion

Line 597: Clarify the type of dossiers referred to in this sentence. Is it for condoms and gloves?

Line 694-708: It is important to reconcile this theory to the study findings in a short preface to this paragraph.

Line 716-717: Italicise for consistency

Line 740: Was this “study” also initiated by the Tanzanian NRA? If so, perhaps make this explicit.

Figure 1: Provide a citation for this figure

Figure 2: Is the roadmap novel in any way or is it adopted entirely from literature? If the latter, there should be a reference provided for the figure.

Overall, the paper's insights into the regulatory challenges and stakeholder perceptions in a resource-limited setting have the potential to resonate with a wide range of individuals and organizations involved in healthcare, policy, research, and advocacy on a global scale. Addressing these weaknesses alluded to above could enhance the overall quality, rigor, and clarity of the report, making it more robust and impactful in contributing to the understanding of medical device regulation in resource-limited settings like Zimbabwe.

Thank you for the opportunity to review this paper.

Yours sincerely,

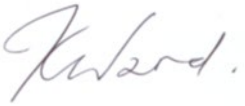

Kim Ward
